# Visual Monitoring Technology for Substation Vulnerable High-Voltage Electrical Equipment Based on ISSA-LSTM Deep Learning Model Coupling Video Overlay Algorithm

**DOI:** 10.1155/2022/3713279

**Published:** 2022-08-26

**Authors:** Shifeng Wang, Xueyong Ding, Qingji Tan

**Affiliations:** ^1^School of Science and Technology, University of Sanya, Sanya 572099, China; ^2^School of Mechanical Engineering, Heilongjiang Agricultural Reclamation Vocational College, Harbin 150025, China

## Abstract

To enhance the visualization effect of substation high-voltage electrical equipment vulnerability, this study proposes an ISSA-LSTM coupled video overlay algorithm-based substation high-voltage electrical equipment vulnerability visualization and monitoring model. Using the improved *α* blending algorithm combined with the inverse sampling of video background color, overlaying visible video as well as infrared video, using the improved adaptive weighted two-dimensional principal component analysis (W2DPCA) to fuse the base layer, selecting the detail layer as the final detail layer, obtaining the final fusion frame, and realizing the visualization and monitoring of substation high-voltage electrical equipment vulnerability, and introducing the improved sparrow search algorithm (ISSA) to establish long and short-term memory network prediction model to reduce the prediction error and improve the monitoring accuracy rate. The experimental results show that the monitoring frames obtained by this method can reflect rich details of substation high-voltage electrical equipment, and the texture color and equipment edge contrast are enhanced to facilitate accurate determination of substation high-voltage electrical equipment vulnerability, and the prediction accuracy of ISSA-LSTM model is as high as 99.85%.

## 1. Introduction

Substation contains many high-voltage electrical equipment such as transformers, circuit breakers, and voltage transformers, which are important components of power system supply and distribution [1]. When a substation encounters a severe vibration situation such as an earthquake, damage to high-voltage electrical equipment will cause the grid to lose its power supply and distribution functions, and power outage conditions enhance the difficulty of resettling victims after a disaster [2, 3]. Substation high-voltage electrical equipment susceptibility can be applied to equipment earthquake risk assessment, and the equipment status determines the operation of substation transmission and distribution systems [4, 5]. Applying visual monitoring technology to the study of substation high-voltage electrical equipment susceptibility can effectively enhance the overall monitoring needs of electrical systems.

The research methods of seismic capacity and vulnerability of high-voltage electrical equipment in substations mainly include theoretical analysis and simulation calculation methods, shaking table test methods, and statistical analysis of earthquake damage, etc. From the 1980s to the present, many scholars at home and abroad have conducted a lot of research on the seismic performance of high-voltage electrical equipment through the first two methods [6]. Theoretical calculations and numerical simulations, shaking table experiments and other methods focus more on the analysis of seismic response of electrical equipment, simulation of equipment damage process, and damage state, which are powerful methods and means to analyze and study the seismic capacity of equipment, damage mechanism, and develop equipment seismic isolation technology [7, 8]. In the 1980s, the Pacific Earthquake Engineering Center (PEER) and Pacific Gas and Electric jointly established the California Substation Equipment Earthquake Damage Performance Database, which recorded the damage data of electrical equipment in 60 230 kV to 550 kV substations during 12 earthquakes in California, and thus mapped the vulnerability curves of electrical equipment, and was widely used in the postdisaster assessment of power systems. The American Applied Technology Council (ATC) gave earthquake vulnerability curves for various types of lifeline equipment and facilities based on expert experience [9] for use on the US earthquake emergency (FEMA) seismic risk analysis system HAZAS [10]. Previously, more research has been conducted for substation visualization and monitoring, and some researchers have studied the design of remote video monitoring system for substations based on self-assembling networks [11]. Designing remote video monitoring system for self-assembled substations, which can remotely monitor the operation status of the equipment in the substation; some researchers proposed to study the one-touch programmed control technology based on video integration and intelligent analysis [12], where video integration technology and intelligent analysis technology are applied to programmed control power systems to improve the control performance of many devices in power systems. Some scholars study intelligent analysis of substation video based on image processing [13] and apply image processing technology to intelligent analysis of substation video to improve the performance of substation equipment status analysis, and all the above studies apply visualization technology to substation equipment monitoring, and all achieve certain results [14, 15]. It can be seen that image fusion techniques are mostly used in relevant visualization and monitoring studies, and the existing image fusion techniques include multisensor information fusion techniques, multiple gray-level image fusion techniques, wavelet transform-based image fusion techniques, etc., but jagged and dark-edge situations occur when the abovementioned image fusion techniques are applied to monitoring the vulnerability of high-voltage electrical equipment in substations [16]. In addition, researchers have also found that visual monitoring alone has some uncertainty and false positives, and there is a strong need for an algorithmic optimization approach to exploit the temporal correlation and reduce feature redundancy of visual monitoring data [17].

In response to the above problems, this study proposes a model based on dimension fusion optimization and long short-term memory (LSTM) [18]. In view of the defect that the input layer weights and hidden layer biases of the network model need manual experience tuning, an improved sparrow is introduced. The search algorithm (SSA) optimizes the detection model [19] and visually monitors the vulnerability of high-voltage electrical equipment in substations through a coupled video overlay algorithm [20]. Therefore, the visual monitoring of the vulnerability of high-voltage electrical equipment in substations based on the ISSA-LSTM deep learning model coupled with the video overlay algorithm can realize the visual monitoring of high-voltage electrical equipment in substations, improve the texture color, edge contrast and richness of details of electrical equipment, and realize high-voltage electrical equipment in substations. Real-time detection of the vulnerability of electrical equipment.

## 2. Construction of Predictive Models

### 2.1. LSTM Network

The LSTM network is a classical network structure in deep learning. The LSTM contributes to model learning at subsequent moments by passing the weight matrix of the implicit layer at different time steps backward in a coefficient-weighted manner through weight parameter conduction [21]. The accumulation of important information and the abandonment of redundant information are achieved through the collaborative work of input, forgetting and output gates with the help of memory units accumulating the weight states of the implicit layer. Long-term memory is achieved by controlling the gradient transformation range through the synergistic work of the memory unit and the gating structure to effectively avoid the problem of gradients disappearing too quickly [22].

### 2.2. SSA and Its Improvements

SSA is a class of heuristic optimization algorithms that simulates the behavior of sparrows foraging and avoiding predators. In SSA, the population is divided into discoverers and followers, and the discoverers are responsible for searching for food in the population space, while the followers follow the discoverers to search the let-go space [23].

SSA is prone to fall into local optimum during iteration and to solve this problem, an improved sparrow search algorithm (ISSA) is proposed in this paper, and the main improvement parts of ISSA are as follows [24]:

(1) The logistic chaos algorithm is borrowed to optimize the population initialization, and the characteristics of chaotic pseudorandomness and ergodicity are used to achieve a better initial global search. In this paper, the pseudorandom sequence is generated with the help of logistic mapping, and the strategy is mathematically formulated as follows:
  (1) $$\begin{array}{c} Z:a_{n+1}=ua_{n}\left(1-a_{n}\right), \end{array}$$
where *Z* is a chaotic variable, and *u* a is a control parameter. When an initial value *a*_0_ is assigned to the chaotic variable, a set of chaotic initial variables can be obtained by iterating through the logic equation with the help of linear mapping, and the linear mapping scheme is as follows:
  (2) $$\begin{array}{c} Z⟶X:X=a+\left(b-a\right)Z. \end{array}$$
(2) In order to better achieve global optimization at the beginning of the iteration and local convergence at the end, this paper proposes an adaptive alert value strategy, which is described as follows:
  (3) $$\begin{array}{c} w=w_{\min}+\left(w_{\max}-w_{\min}\right)\times \tan\left(\frac{k}{M}\right)\times \frac{\pi }{4}. \end{array}$$
When the current number *k* of iterations is small, *w* is close to *w*_min_ to ensure the global let-down capability of the intelligent algorithm, and as the number of iterations increases, *w* increases in a nonlinear manner to ensure better local let-down convergence in the later iterations, thus allowing the algorithm to flexibly adjust the global let-down and local search capability, that is, *M* is the total number of iterations.
(3) The adaptive mutation factor is introduced by borrowing the idea of mutation from genetic algorithm. After each iteration is completed, the population has a certain chance to mutate, and the mathematical formulation of the strategy is as follows:
  (4) $$\begin{array}{c} p=0.5-\frac{k}{2M}, \end{array}$$
where *p* is the variation factor, and the probability of population variation decreases as the number of iterative generations increases.
(4) The original movement method is the main reason for standard SSA to fall into local optimum when alerting occurs during reconnaissance. In this paper, we choose a new sparrow change method with the following mathematical formulation:
  (5) $$\begin{array}{c} x_{i,d}^{t+1}=\left\{\begin{array}{cc} x_{i,d}^{t}+\beta \cdot \left(x_{i,d}^{t}-xb_{i,d}^{t}\right), & f_{i}\neq f_{g},\\ x_{i,d}^{t}+\beta \cdot \left(xw_{i,d}^{t}-xb_{i,d}^{t}\right), & f_{i}=f_{g}. \end{array}\right. \end{array}$$

*β* is the correlation coefficient, and *f*_*i*_ and *f*_*g*_ are the parameter values of different forms. When the population produces warning behavior, the optimal sparrow will flee to a random position between the optimal and worst positions, and the remaining sparrows will flee to a random position between themselves and the worst position.

### 2.3. Video Overlay Algorithm Design

The captured visible substation video as well as the infrared substation video are superimposed using an improved *α*-blending algorithm combined with background color inverse walk [25]. The so-called “*α*-blending” algorithm is used to blend the source and target pixels using *α*-blending vector values to create a sense of transparency in 3D objects. The *α*-blending algorithm is introduced in this study to give a three-dimensional sense of high-voltage electrical equipment [26]. The *α*-blending algorithm weighted summation of video near and far views based on a fixed ratio is given by the following equation:

(6) $$\begin{array}{c} I_{a}=\alpha I_{l}+\left(1-\alpha \right)I_{r}. \end{array}$$

In (6), *α* and (1 − *α*) denote the near-field weights and the corresponding far-field weights in the video, respectively; *I*_*l*_ and *I*_*r*_ denote the near-field pixel points and the corresponding far-field pixel colors, respectively, *I*_*a*_ denotes the output value after superimposing the video using the *α* mixing algorithm.

Based on the grayscale, coordinates and color of the pixel points obtained by the pixel desampling algorithm, the desampling equation is implemented for the corresponding locations in the video far field as follows:

(7) $$\begin{array}{c} I_{b}=I_{r}+\left(I_{e}-I_{r}\right)J_{g}. \end{array}$$

Equation (7), *I*_*b*_ that the body of the video screen and the distant field on the antisample pixel color; *I*_*e*_ and *J*_*g*_ that the distant field pixels are located in the same position in the near field corresponding pixel color and based on the corresponding antisample to obtain the near field pixel gray level.

The new near-field screen is obtained through (7), and the new screen is based on the far-field basis to implement antisampling to avoid black edges and jagged boundaries between the far-field and near-field. The acquired near-field and far-field can be well integrated, but the background color in the original screen and the far-field fusion leads to the alpha channel cannot be mixed using the background color detection, the transformation formula (7) is as follows:

(8) $$\begin{array}{c} I_{b}=\left\{\begin{array}{c} I_{l},I_{l}=I_{k},\\ I_{r}+\left(I_{e}-I_{r}\right)J_{g},I_{l}\neq I_{k}. \end{array}\right. \end{array}$$

Formula (8), *I*_*k*_ is the original video screen background color.

Using the above process to obtain a mix of channel *α*, the inverse walk-through on the local video screen of the telepresence to achieve the mix formula is as follows:

(9) $$\begin{array}{c} I_{out}=\left\{\begin{array}{cc} I_{r}, & I_{b}=I_{k},\\ \alpha I_{b}+\left(1-\alpha \right)I_{r}, & I_{b}\neq I_{k}. \end{array}\right. \end{array}$$

In (9), indicates the final video overlay output value.

Based on the background color to determine the channel mix within the channel, the implementation of inverse sampling of the distant screen contains the original background color and the new pixel points, the new background color is also presented in the video screen.

The new background color within the channel discriminant may not be discriminated to be located within the image element, which can be transformed into (9) as follows:

(10) $$\begin{array}{c} I_{\text{out}}=\left\{\begin{array}{cc} I_{r}, & I_{l}=I_{k},\\ \alpha I_{l}+\left(1-\alpha J_{g}\right)I_{r}, & I_{l}\neq I_{k}. \end{array}\right. \end{array}$$

In (10), *I*_*out*_ indicates the final video overlay result of the channel mix.

Analysis of the above process shows that when the algorithm is used to superimpose the video, the blending weights are transformed from fixed values to variable blending parameters, and the corresponding far-field weights of the variable blending parameters can be adjusted according to the gray level of the near-field pixel points at each position, and the far-field within the blending weights are smaller when the near-field gray level is higher, and the far-field within the blending weights are larger when the near-field gray level is lower, resulting in the following formula:

(11) $$\begin{array}{c} I_{\text{out}}=\left\{\begin{array}{cc} I_{r}, & I_{l}=I_{k},\\ I_{a}+\left(1+J_{g}\right)\alpha I_{r}, & I_{l}\neq I_{k}, \end{array}\right.\\ \eta =\left(1-J_{g}\right)\alpha ,\\ I_{\text{out}}=\left\{\begin{array}{cc} I_{r}, & I_{l}=I_{k},\\ I_{a}+\eta I_{r}, & I_{l}\neq I_{k}. \end{array}\right. \end{array}$$

Equation (11) shows that the algorithm adds the correction function to the traditional *n*-blending algorithm and sets the blending weights to compensate for the correction value based on the near-field gray level so that the blended video does not have jaggedness and dark edges after superposition.

When the near-field blending weight is large and the gray level of the pixel is fixed, and the gray level of the blended pixels involved in the superposition is low, the far-field of the superposition will be covered by the near-field, and the larger the near-field blending weight is, the more obvious the jaggedness and dark edges will be, so it is necessary to increase the correction amount to compensate for this. When the gray level of the pixel points in the near-field superposition is too low and the blending weight is fixed, the jaggedness and dark edges exist at the boundary of the superposition element when the far-field blending weight is not the maximum value, and the gray level of the near-field pixel points is negatively correlated with the correction amount.

The video overlay algorithm is applied to the established substation model. This is when the video realizes the visual monitoring of the vulnerability of high-voltage electrical equipment in the substation, and the pixel information of the close-view screen existing in RGB space does not have gray-level information. RGB space data are displayed using YUV space gray-level information with the following conversion formula:

(12) $$\begin{array}{c} Y=0.299R+0.587G+0.114B, \end{array}$$

where *R* and *B* are both parameters of the equation.

### 2.4. Fusion Algorithm Based on Bootstrap Filter and W2DPCA

#### 2.4.1. Visual Surveillance Video Frame Decomposition with Bootstrap Filters

The fusion of visible video and infrared video after video overlay to obtain higher-quality visual surveillance results so that the visual surveillance video is not affected by the surrounding environment, the adaptive weighted two-dimensional principal component analysis (hereinafter referred to as adaptive W2DPCA algorithm) is combined with the bootstrap filter to fuse visible video as well as infrared video, and this fusion method has the advantage of low-computational complexity and is able to obtain the visual surveillance results by the frame-by-frame fusion. This fusion method has the advantage of low-computational complexity and is able to obtain visual surveillance results by fusing visible and infrared video on a frame-by-frame basis [27]. The fusion method mainly consists of dividing the visual monitoring layers by using the guidance filter, obtaining the base and detail layers of the visible and infrared frames monitoring results, selecting different planning methods to fuse the base and detail layers obtained by video layering, and finally combining the fused base and detail layers to obtain the final substation high-voltage electrical equipment vulnerability visual monitoring results:

(13) $$\begin{array}{c} I_{B}^{i}=G_{r,\xi }\left(I^{i},I^{i}\right),\\ I_{B}^{v}=G_{r,\xi }\left(I^{v},I^{v}\right). \end{array}$$

In the above equations, *G* and *r* denote the bootstrap filter function and the filter radius, respectively; *I*^*i*^ and *I*^*v*^ denote the source infrared frame and the source visible frame, respectively; *ξ* and *I*_*B*_^*v*^ denote the regularization parameter and the visible frame base layer, respectively; *I*_*B*_^*i*^ denotes the infrared frame base layer.

The visible frame and the infrared frame detail layer are obtained using the difference between the visible base layer and the infrared base layer obtained from the source visible frame and the source infrared frame [14]. The obtained infrared frames and visible detail layers are given by

(14) $$\begin{array}{c} I_{D}^{i}=I^{i}-I_{B}^{i},\\ I_{D}^{v}=I^{v}-I_{B}^{v}. \end{array}$$

The base and the detail layers obtained by the above process retain the large variance region and texture information within the source video, respectively, and the target within the infrared image exists within the video base layer.

#### 2.4.2. W2DPCA Fusion Base Layer

The quality of video frames directly affects the final result of image fusion [28], and only infrared thermal images need to be added to high-quality visible frames to obtain the best fusion results. The process of fusing visible frames with IR frames using the adaptive W2DPCA algorithm is as follows:

The W2DPCA algorithm is first implemented on the infrared frame and visible frame base layer, and the set of infrared frames and visible frames is represented by matrix *P*. The matrix *P* covariance matrix *G* is obtained as follows:

(15) $$\begin{array}{c} G=\frac{1}{2}\sum_{i=1}^{2}{\left(P^{i}-\mu \right)}^{T}\left(P^{i}-\mu \right). \end{array}$$

In Equation (19), (*P*^1^) denotes the infrared frame, *P*^2^ and *u* denote the visible frame as well as the average of visible and infrared frames, respectively.

The eigenvectors of the covariance matrix *G* are arranged in descending order according to the size of the eigenvalue *λ*. The eigenvectors corresponding to the largest eigenvalue are placed on the leftmost side of the matrix, using *U* to represent the extracted Eigenmatrix, and the Eigenmatrix of size *n* × *d* is composed of the first *d* eigenvectors, and the matrix expression is as follows:

(16) $$\begin{array}{c} U=\left(U_{1},U_{2},\ldots ,U_{d}\right). \end{array}$$

The equations for obtaining the feature image *Q*_*B*_^*i*^ and *Q*_*B*_^*v*^ are as follows:

(17) $$\begin{array}{c} Q_{B}^{i}=I_{B}^{i}\times U=\left[Q_{B_{1}}^{i},Q_{B_{2}}^{i},\ldots ,Q_{B_{d}}^{i}\right],\\ Q_{B}^{v}=I_{B}^{v}\times U=\left[Q_{B_{1}}^{v},Q_{B_{2}}^{v},\ldots ,Q_{B_{d}}^{v}\right]. \end{array}$$

The feature image consists of the infrared frame and the first *d* principal components of the photon frame. The different frame weights change as the pixel size changes. The visible light frame and infrared frame weights are calculated by using the feature image with smaller size, which can effectively reduce the calculation amount.

The fusion process needs to detect whether there is a difference in contrast between the edge regions of the visible light frame and other regions [29]. The quality of visible light is reduced in poor environments, but still contains useful texture information. Fully considering the frame quality to obtain adaptive visible light weights in the fusion process, the visible light frame weight formula based on regional variance is as follows:

(18) $$\begin{array}{c} W^{v}=\delta^{2}=\frac{1}{n\times d}\sum_{i=1}^{n}\sum_{j=1}^{d}{\left(Q_{B}^{v}\left(i,j\right)-\bar{Q_{B}^{v}}\right)}^{2}. \end{array}$$

In formula (18), *δ* represents the standard deviation of the source image. Higher and lower pixel values in the infrared frame indicate hotter and cooler regions, respectively. The grayscale values of all pixels in the infrared frame are significantly lower in the low-temperature case than in the high-temperature case, and the grayscale values of hot objects in the image are significantly higher than those of other pixels. The fusion process should focus on the infrared thermal objects, using the zero-mean operation to obtain the infrared weights Wi equation as follows:

(19) $$\begin{array}{c} W^{i}=\delta^{2}=\frac{1}{n\times d}\sum_{i=1}^{n}\sum_{j=1}^{d}{\left(Q_{B}^{i}\left(i,j\right)-\bar{Q_{B}^{i}}\right)}^{2}. \end{array}$$

It can be seen by (19) that the larger weights will be assigned to the higher pixel fraction.

Using the weighted average method to obtain the fused feature image *Q*_*B*_ of infrared frames as well as visible frames, the fused feature map can retain all the detail information of visible frames by this method, and the fused feature map equation is as follows:

(20) $$\begin{array}{c} Q_{B}=\frac{W^{v}Q_{B}^{v}+W^{i}Q_{B}^{i}+Q_{B}^{v}}{W^{v}+W^{i}+1}. \end{array}$$

The following equation is used to approximate the reconstruction of the fusion base layer equation as follows:

(21) $$\begin{array}{c} I_{B}=Q_{B}\times U^{T}. \end{array}$$

#### 2.4.3. Detail Fusion Layer

The real texture information within the image may destroy the large amount of useless information contained in the IR frame detail layer, and the IR frame image detail layer may be discarded during the fusion process, and the detail layer fusion equation is as follows:

(22) $$\begin{array}{c} I_{D}=I_{D}^{v}. \end{array}$$

#### 2.4.4. Final Fused Video Frames

The final fusion formula for the fused base layer and the fused detail layer is as follows:

(23) $$\begin{array}{c} I_{F}=I_{B}+I_{D}. \end{array}$$

The final fusion frame obtained by (23) can effectively represent the thermal target and texture information contained in the infrared frame as well as the visible frame.

## 3. Modeling Method

### 3.1. Data Preprocessing

To prevent the loss of low order of magnitude features, this paper uses a normalization strategy to preprocess the original features, and the original set of features is normalized to the data segment of [0, 1], and the normalization mathematics is expressed as follows:

(24) $$\begin{array}{c} x=\frac{x-x_{\min}}{x_{\max}-x_{\min}}, \end{array}$$

where *x*_max_ is the maximum value in the sample features and *x*_min_ is the minimum difference in the sample features.

### 3.2. Model Training

A total of 70% of the data were selected as training model and 30% as validation model. PCA is used to process the input data to achieve data dimension reduction, and the input format of data is processed by sliding window to train the LSTM detection model.

### 3.3. Intelligent Algorithm Optimization and Its Improvement

Compared with other swarm intelligence optimization algorithms, SSA has high search accuracy, fast convergence, good stability, and strong robustness. However, when SSA search approaches global optimum, the population diversity will decrease and fall into local optimum. In this paper, SSA is improved and ISSA algorithm is proposed. According to the fan data modeling requirements, the search dimension was set as 3D, the population size was 20, and the number of iterations was 1000. Different test functions were selected for testing, and the results were shown in Table 1.

### 3.4. Prediction Accuracy

PrecisionRecall curve derived from the evaluation of correlation in information retrieval was used to reflect the accuracy of fault decision of icing model, that is, recall rate and accuracy:

(25) $$\begin{array}{c} \text{recall}=\frac{TP}{TP+FN},\\ \text{precision}=\frac{TP}{TP+FP},\\ F_{1}=2\times \frac{\text{precision}\cdot \text{recall}}{\text{precision}+\text{recall}}. \end{array}$$

In the formula, *T*/*F* represents whether the predicted result is consistent with the actual situation: that is, if the real situation is a positive sample (*P*) and the prediction is a positive sample (*P*), it is *T*; if the real situation is a negative sample (*N*), the prediction is Negative samples (*N*), it is *T*; if the true case is *P*, the prediction is *N*, it is *F*; if the true case is *N*, the prediction is *P*, it is *F*. That is, TP is a positive sample that is actually a positive sample; FN is a negative sample that is actually a positive sample; FP is a positive sample that is actually a negative sample. Recall represents recall rate, precision represents precision, and fraction represents harmonic mean of precision rate and recall rate.

## 4. Results' Analysis

### 4.1. Video Overlay Algorithm Analysis

In order to verify the effectiveness of the visualization and monitoring of substation high-voltage electrical equipment susceptibility based on video overlay algorithm for the study of substation high-voltage electrical equipment susceptibility, a power system composed of a national grid is selected as an example analysis object, which is characterized by a large amount of industrial electricity consumption and high population density. A total of 29 substations above 110 kV in the region were selected as the sample for the statistical analysis of high-voltage electrical equipment vulnerability study: 2 500 kV substations, 8 220 kV substations, and 19 110 kV substations. Design a set of substation high-voltage electrical equipment vulnerability visualization monitoring system, the system uses infrared sensors and visible sensors of online monitoring devices to obtain substation high-voltage electrical equipment image source, using the upper computer to run the system using the method of this paper to process the collected images, through the C language using VS compiler platform programming this paper software, the system has infrared image and visible image fusion, infrared and visible imaging information complementary, substation high-voltage electrical equipment vulnerability analysis, manual operation, and other functions. This method uses the video overlay method to superimpose the video so that the presented video has no jaggedness; the visual monitoring video has the equipment perspective function, which can set the transparency of each high-voltage electrical equipment in the substation, so as to avoid the inability to accurately analyze the vulnerability of the equipment due to some equipment being obscured. A pair of infrared frames and visible frames are selected from the video of high-voltage electrical equipment in the source substation, and the objective indexes of the image quality before and after the fusion process are shown in Table 1, using this method to superimpose the above two original images.

The index data in Table 2 show that this method can fuse outdoor substation scenes well, and the fused images can show the texture of high-voltage electrical equipment in the visible video and the equipment information in the infrared video well, and the edge performance is more natural, and the visualization effect of the fused video is significantly improved compared with that before the fusion, and the shadow part of high-voltage electrical equipment is enhanced, and the contrast is improved, and the details of the equipment can be strengthened effectively in the fusion result, and the color of the equipment texture and the contrast of the equipment edge are enhanced, and the overall details of the high-voltage electrical equipment in the monitoring video of the substation are richer.

Based on the three-dimensional model of the substation established in this paper, Matlab simulation software is used to simulate a real earthquake disaster, given that the nodes sampled by the substation information are 200, the maximum daily load is 120 kW, the active power of the substation is 42 kW, and the simulated earthquake disaster level is 6.7. Due to the difference between different regions and the distance from the earthquake source, the damage of high-voltage electrical equipment in different regions is different, and the distance between the substation and the earthquake source is used as the standard to divide the studied region into six regions from A to F. Based on the above settings, the damage of high-voltage electrical equipment in each region is shown in Table 3.

The results in Table 3 show that the number of damaged devices decreases as the distance from the source increases, which are consistent with reality and fully verifies the effectiveness of the simulation of high-voltage electrical equipment in the substation. Based on the visualization and monitoring interface, it can be seen that the main damage states of the porcelain column-type equipment in the substation, except for transformers, are fracture damage and crack damage. Equipment fracture damage affects the use of equipment, equipment cracks do not affect the continued use of equipment cracks set to undamaged, the equipment fracture state is classified as complete damage. Transformer damage state in the earthquake is mainly manifested as wheel rail fixing device damage, oil pillow damage, and other component damage, it can be seen that the transformer has a low vulnerability. Transformer damage can be divided into two states: damaged parts and undamaged parts, and transformer parts are undamaged when the damage to the parts is less than one-fifth of the transformer itself and does not affect the normal operation of the transformer; otherwise, the transformer parts are damaged.

### 4.2. SSA-LSTM Prediction Optimization Analysis

**Figure 1:**
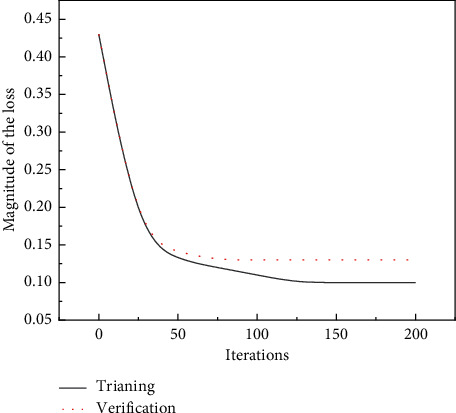
LSTM model training loss.

The sliding window width is set to 10, the learning rate of the optimizer is 0.001, the number of neurons in the hidden layer is 50, the step size parameter is 64, the model is trained 200 times, and the Adam optimizer is selected to optimize the model. When the training samples are 20,000, the LSTM model training loss is shown in Figure 1. In Figure 1, the dashed line represents the loss variation of the model validation set, and the solid line represents the loss variation of the training set. Analysis of the curves in the figure shows that the training loss of the LSTM model has converged to around 0.13 at about 105 iterations. The samples after feature fusion processing were selected as training data to construct ISSA-LSTM model and compared with the optimized models of other swarm intelligence optimization algorithms to verify the optimization of ISSA, SSA, PSO, and GWO on the detection model, and the model validation set accuracy rates are shown in Table 4.

**Figure 2:**
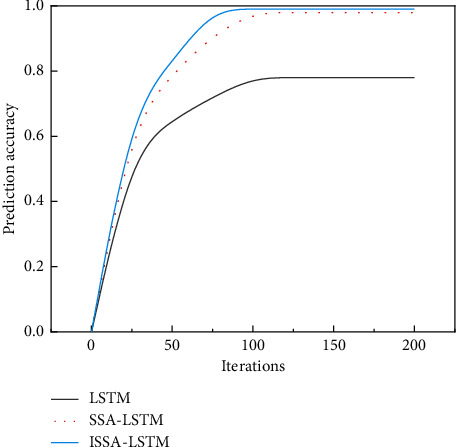
The relationship between the number of iterations and model accuracy.

From Figure 2 and Table 4, it can be seen that the LSTM and its coupled models ISSA-LSTM, SSA-LSTM, PSO-LSTM, and GWO-LSTM are in a stable state after reaching a certain number of training sessions and slowly improve, increasing with the number of training sessions. However, it is found that the prediction effect of LSTM model is significantly lower than its coupled model, and the prediction accuracy of LSTM model is 97.77% after 20,000 training times, while the prediction accuracy of PSO-LSTM model, which is the worst prediction effect among the coupled models, is 99.07, which is significantly higher than that of LSTM model. In addition, the improved SSA model, whose improved model ISSA coupled with LSTM model has the highest prediction accuracy of 99.85% after 20,000 training cycles, which indicates that the LSTM model alone has a certain prediction deficiency, probably due to its own model factors, but coupling it with other model algorithms can significantly improve its prediction accuracy and prediction effect.

In addition, this study takes 20,000 training samples, and the rest parameters are kept consistent for the experiments, and the parameters of the group intelligence algorithm optimization model are shown in Table 5. Compared with SSA, PSO, GWO, and other group intelligence optimization algorithms, the parameters of ISSA are better than those of other group intelligence algorithms, and the judgment accuracy of 99.85% can be achieved when the training sample data is 20,000, which fully proves the effectiveness and reliability of ISSA-LSTM model. Meanwhile, the *F*1 scores of both positive and negative samples show a decreasing trend. However, the accuracy and recall rates of ISSA and GWO are extremely close, which indicates that GWO may be able to be used as a potential coupled analysis model to enhance the prediction effect of the LSTM model.

## 5. Conclusion

The ISSA-LSTM coupled video overlay algorithm is applied to the visual monitoring of high-voltage electrical equipment vulnerability in substations. The video overlay algorithm can avoid the jaggedness and dark edges of the borders of the close view of the overlaid video images, which meet the practical requirements of high-voltage electrical equipment vulnerability monitoring in substations. The researched method has high 3D visualization monitoring performance, strengthens the details of electrical equipment, enhances its texture color and edge contrast, improves the richness of electrical equipment details in the monitoring video, accurately analyzes the vulnerability of high-voltage electrical equipment using substation visualization monitoring, and improves the safe operation of substations. The following conclusions were obtained: (1) the images after the video overlay algorithm can show the texture of high-voltage electrical equipment in the substation in the visible video and the information of the equipment in the infrared video well, and the edge performance is more natural. (2) The prediction effect of the LSTM model is significantly lower than that of its coupled model, and the prediction accuracy of the LSTM model is 97.77% after 20,000 times of training, while as the coupled prediction accuracy of PSO-LSTM model, which has the worst prediction effect, is 99.07, which is significantly higher than that of LSTM model. (3) Compared with SSA, PSO, GWO, and other group intelligence optimization algorithms, the parameters of ISSA are better than other group intelligence algorithms, and the judgment accuracy of 99.85% can be achieved when the training sample data are 20,000.

## Figures and Tables

**Table 1:** Benchmark function results.

Benchmark function	Dimension	Search space	Optimal value	SSA	ISSA
*F*_1_(*x*)=∑_*i*=1_^*n*^*x*_*i*_^2^	3	[−100, 100]^*n*^	0	4.006 × 10^−3^	4.006 × 10^−89^
*F*_2_(*x*)=∑_*i*=1_^*n*^|*x*_*i*_|+∏_*i*=1_^*n*^|*x*_*i*_|	3	[−100, 100]^*n*^	0	1.049 × 10^−1^	6.023 × 10^−17^
*F*_3_(*x*)=∑_*i*=1_^*n*^(∑_*i*=1_^*i*^*x*_*j*_)^2^	3	[−100, 100]^*n*^	0	2.891 × 10^−1^	1.343 × 10^−89^
*F*_4_(*x*)=max_*i*_{|*x*_*i*_|, 1 ≤ *i* ≤ *n*}	3	[−100, 100]^*n*^	0	1.779 × 10^−1^	1.624 × 10^−46^
*F*_5_(*x*)=∑_*i*=1_^*n*−1^[100(*x*_*i*+1_ − *x*_*i*_^2^)^2^+(*x*_*i*_ − 1)^2^]	3	[−30, 30]^*n*^	0	3.262 × 10^−2^	1.0667 × 10^−3^

**Table 2:** Objective index comparison of image quality before and after fusion.

Objective indicators	Prefusion results	Postfusion result
Entropy	7.55	7.57
Standard deviation	55.39	56.48
Root mean square error	6.63	2.84
Peak signal-to-noise ratio	70.36	87.33
Spatial frequency	11.026	15.51
Average gradient	5.192	6.09

**Table 3:** Degree of damage to high-voltage electrical equipment.

Divide area	Substation	Total number of high-voltage electrical equipment	Quantity of damaged equipment
A	6	243	197
B	4	185	115
C	6	285	97
D	5	219	76
E	4	197	58
F	4	193	39

**Table 4:** Accuracy of swarm intelligence algorithm model.

Training set	LSTM	ISSA-LSTM	SSA-LSTM	PSO-LSTM	GWO-LSTM
1000	93.25	95.24	93.96	93.37	94.31
2500	95.45	97.59	97.11	96.17	96.44
5000	96.64	98.33	97.23	96.93	97.14
10,000	97.50	99.36	98.69	98.33	98.48
15,000	97.74	99.67	99.26	99.07	98.90
20,000	97.77	99.85	99.46	99.16	99.49

**Table 5:** Comparison of optimized parameter results.

Swarm intelligence optimization algorithm	Positive sample judgment accuracy rate (%)	Negative sample judgment accuracy rate (%)	Positive sample recall rate (%)	Negative sample recall rate (%)	Positive sample *F*1 score	Negative class sample *F*1 score
ISSA	99.89	99.81	99.38	99.96	0.996	0.998
SSA	99.58	99.40	98.55	99.84	0.990	0.996
PSO	99.47	99.03	97.63	99.80	0.985	0.994
GWO	99.89	99.34	98.50	99.96	0.991	0.996

## Data Availability

The dataset can be obtained from the corresponding author upon request.
